# Long-Term Regulation of IL-17 Expression in Pacific Oyster Hemocytes by mGluR5 Through the Phosphoinositide Pathway

**DOI:** 10.3390/cells14060438

**Published:** 2025-03-14

**Authors:** Yiran Si, Deliang Li, Wenjing Ren, Xueshu Zhang, Lingling Wang, Linsheng Song

**Affiliations:** 1 Liaoning Key Laboratory of Marine Animal Immunology & Disease Control, Dalian Ocean University, Dalian 116023, China; siyrxxx@163.com (Y.S.); l17685898516@163.com (D.L.); rwj1012423@163.com (W.R.); lshsong@dlou.edu.cn (L.S.); 2 Liaoning Key Laboratory of Marine Animal Immunology, Dalian Ocean University, Dalian 116023, China; 3 Dalian Key Laboratory of Aquatic Animal Disease Prevention and Control, Dalian Ocean University, Dalian 116023, China; 4 Southern Marine Science and Engineering Guangdong Laboratory (Zhuhai), Zhuhai 519000, China

**Keywords:** glutamate, *mGluR5*, *Crassostrea gigas*, hemocyte, *IL-17* expression

## Abstract

Metabotropic glutamate receptor 5 (mGluR5) is a critical regulator of immune responses within the neuroimmune system, influencing cytokine secretion and immune cell function. Although extensively studied in mammals, its role in regulating IL-17 in invertebrate immunity is poorly understood. This study examines *CgmGluR5* expression and downstream signaling activation in Pacific oyster (*Crassostrea gigas*) hemocytes following glutamate (Glu) and *Vibrio splendidus* treatment. Glu treatment significantly induced the expression of *CgmGluR5* and key signaling molecules, including PLC, DAG, IP3, Ca²⁺, and PKC, while enhancing mRNA levels of *CgIL17-1*, *CgIL17-5*, and *CgCaspase3*. Elevated Ca²⁺ content and *CgIL17* expression in hemocytes were observed at 12 h post-Glu exposure, indicating *CgmGluR5*-mediated immune regulation through the phosphoinositide pathway. A 1.14-fold increase in the apoptosis rate was found in the Glu treatment group compared to the control group. Knockdown of *CgmGluR5* suppressed *CgIL17-1* and *CgIL17-5* expression and reduced granulocyte proportions, reflecting its role in immune regulation. This study shows that *CgmGluR5* mediates long-term immune regulation in oysters through the phosphoinositide pathway, providing new theoretical insights for aquaculture immune management.

## 1. Introduction

Metabotropic glutamate receptors (mGluRs), notably mGluR5, are key components of the neuroendocrine–immune (NEI) system, bridging neural and immune functions [1,2]. In vertebrates, mGluR5 modulates immune responses through diverse mechanisms, including cytokine production, cell proliferation, inflammatory signaling, and apoptosis regulation through pathways such as the Gq protein and β-arrestin-mediated cascades [3,4]. These functions are critical for immune homeostasis and inflammatory responses [5]. mGluR5 also facilitates neuroimmune interactions, promoting proinflammatory cytokine release by microglia and calcium signaling in glial cells [6,7,8]. However, despite extensive studies in vertebrates, the role of *mGluR5* in invertebrate immune systems remains largely unexplored. Elucidating the mechanisms of mGluR5 in invertebrate immunity may reveal conserved immune functions and provide novel insights into marine immune responses.

Interleukin 17 (IL-17) is a proinflammatory cytokine pivotal to immune regulation and inflammatory responses across vertebrates and invertebrates [9,10]. In mammals, IL-17 promotes immune responses by inducing cytokine production, recruiting immune cells, and facilitating pathogen clearance [9,11,12]. Homologs of IL-17 have been identified in invertebrates, including oysters, emphasizing its role in innate immunity [13,14]. However, the regulatory mechanisms of IL-17 expression in oysters remain insufficiently characterized, particularly in relation to glutamate receptor signaling. Evidence suggests that *Cg*mGluR5 modulates immune gene expression and calcium signaling pathways in hemocytes, potentially influencing IL-17 expression and immune cell activity [2]. Investigating this regulatory interaction will deepen our understanding of immune signaling pathways in oysters.

The Pacific oyster (*Crassostrea gigas*) presents an ideal model for studying immune regulation due to its ecological and economic relevance and exposure to pathogen-rich environments [15]. Unlike vertebrates, oysters rely solely on innate immune mechanisms to combat microbial infections, with hemocytes serving as key players in pathogen recognition and elimination [16,17]. Studying *Cg*mGluR5-mediated immune responses, particularly through phosphoinositide signaling and its impact on IL-17 regulation, offers valuable insights into the molecular mechanisms underpinning immune defense in marine mollusks. This research contributes to a foundational understanding of glutamate-mediated immune regulation in invertebrates, offering a scientific basis for enhancing immune management strategies in aquaculture.

## 2. Materials and Methods

### 2.1. Experimental Animals and Microorganisms

This study selected Pacific oysters with a shell length of 10 ± 5 cm, sourced from aquaculture farms in Dalian, Liaoning Province, China. Prior to the experiments, all oysters were maintained in aerated seawater tanks at a temperature of 15–20 °C for over a week. All experiments were conducted in compliance with the animal ethics guidelines approved by the Ethics Committee of Dalian Ocean University. The microorganism used in this study was *V. splendidus*, which was preserved in the laboratory and cultured in 2216E medium at 28 °C [18].

### 2.2. Experimental Treatments and Sample Collection

This study used two-year-old Pacific oysters collected from a Dalian aquaculture farm and acclimated to aerated, filtered seawater at 22 °C for one week. They received controlled daily water changes and spirulina feeding to minimize environmental variability. *V. splendidus* was cultured to an OD600 of 0.6 and injected at 2 × 10^8^ CFU/mL into oysters in the experimental group, while control oysters received sterile seawater. Hemocyte samples were collected at 0, 3, 6, 12, 24, 48, and 72 h post-injection with biological replicates at each time point. In the glutamate (Glu) treatment experiment, oysters were immersed in seawater containing 10^−4^ M Glu, with hemocytes sampled at intervals (0, 3, 6, 12, and 24 h). In the *CgmGluR5* inhibitor treatment experiment, 54 oysters were equally divided into three groups. The first group was injected with 100 μL of sterile seawater per oyster as the SW group. The second group was injected with 100 μL of a 500 nM MPEP (MCE, dissolved in sterile seawater) solution per oyster as the MPEP group. Sampling was performed at 0, 6, and 12 h post-stimulation. For PKC and PLC inhibition experiments, three groups were prepared: a control group (seawater injection), a group treated with D609 (PLC inhibitor, 10.0 μmol/L), and a group treated with Bisindolylmaleimide I (PKC inhibitor, 10.0 μmol/L), and hemocyte samples were collected 12 h post-injection. Due to the lack of a specific antibody, we assessed RNAi efficiency by measuring mRNA expression levels. For RNA interference experiments, dsRNA expression vectors were constructed and introduced into RNase III-deficient Escherichia coli HT115 (DE3). The dsRNA fragments of *CgmGluR5* and EGFP were induced by IPTG. RNA interference experiments involved injecting oysters with EGFP (used as a control to ensure that the dsRNA injection did not induce non-specific effects and to validate the specificity of *mGluR5* inhibition) or dsRNA targeting *CgmGluR5*, with hemocyte samples taken at 0 and 12 h [1]. In each experimental group, 9 animals were used, with each sample divided into 3 groups of 3 animals. The 6–12 h time points were chosen to capture long-term metabolic effects, as immune cells like hemocytes undergo adaptation and metabolic reprogramming over time. Short time points represent more immediate signaling responses. Samples were homogenized using TRIzol for RNA extraction, with rigorous data pre-processing to ensure consistency. Randomization minimized selection bias, while variable controls and repeatability measures, including consistent sampling intervals and ELISA quantification of signaling molecules, assured robust and reproducible results.

### 2.3. Quantitative RT-PCR Analysis

Total RNA was extracted from hemocytes with TRIzol reagent following the instructions provided by the manufacturer [19]. cDNA was synthesized from RNA using the TransScript One-Step gDNA Removal and cDNA Synthesis Kit (TransGen Biotech, Beijing, China). Quantitative real-time PCR (RT-qPCR) was performed using the PrimeScript™ RT Master Mix on a Light Cycler 7500 real-time PCR system (Applied Biosystems^®^, Carlsbad, CA, USA). The expression levels were normalized to *CgEF-1*. Relative expression levels of target genes were analyzed via the 2^−ΔΔCT^ method. The corresponding primer sequences used for RT-qPCR are listed in Supplementary Table S1.

### 2.4. Protein Abundance Analysis Using Western Blotting

Total proteins from hemocytes were extracted using RIPA lysis buffer, as described previously [20]. After separation by SDS-PAGE, proteins were transferred to a nitrocellulose membrane using an electrophoretic transfer apparatus. The membrane was incubated with 1% skim milk at 37 °C for 3 h, followed by overnight incubation with primary antibodies (1:1000 dilution) at 4 °C. The membrane was then incubated with HRP-conjugated secondary antibodies (goat anti-rabbit or goat anti-mouse IgG, 1:1000, Proteintech) at 37 °C for 1 h. After being heavily washed three times with TBST, the membranes were finally incubated with SuperSigna ECL Western blot substrates for 30 s and imaged with Amersham Imager 600. The other antibodies used for Western blotting were monoclonal antibodies, including mouse monoclonal antibodies against pERK (1:1000, Cell Signaling Technology, Boston, MA, USA) and HRP-conjugated rabbit monoclonal antibodies against beta-Tubulin (1:1000, Proteintech, Chicago, IL, USA).

### 2.5. Hemocyte Typing Analysis

Hemocytes collected from nine oysters in the Glu + dsEGFP and Glu + dsmGluR5 groups were immediately fixed with 4% paraformaldehyde at room temperature for 15 min. They were then used for flow cytometry analysis. Based on previous studies, the hemocyte subpopulations (agranulocyte, semi-granulocyte, and granulocyte) were distinguished according to their size (forward scatter, FSC), internal complexity (side scatter, SSC), and percentage [21]. Flow cytometry was used to analyze changes in the total hemocyte percentages of the three subpopulations.

### 2.6. Hemocyte Apoptosis Detection

Hemocytes collected from nine oysters in the SW and Glu groups were analyzed for apoptosis using the Annexin V-FITC apoptosis detection kit (Beyotime Biotechnology, China) in accordance with the manufacturer’s instructions. Flow cytometry (FCM) with Annexin V-FITC/PI dual staining was used to determine the apoptosis rate. Hemocytes were centrifuged at 800× *g*, 4 °C for 10 min, and then washed in fresh modified L-15 medium. Following the instructions provided by the manufacturer, 195 μL of diluted hemocytes (final concentration of 5 × 10^5^–10^6^ cells/mL) were incubated with 5 μL of Annexin V-FITC in the dark for 10 min to label early apoptotic cells. Then, 10 μL of propidium iodide (PI) was added and incubated for 5 min to label late apoptotic or necrotic cells. The apoptosis rate was measured using a flow cytometer (BD FACS Aria II SORP) [22].

### 2.7. Glutamate Content Measurement

The glutamate (Glu) content in oyster serum was measured according to the instructions of the Glutamate ELISA Kit (Mlbio, Shanghai, China). The procedure was as follows: 50 μL of serum and 50 μL of HRP-labeled Glu antibody were added to a 96-well plate coated with anti-Glu antibodies and incubated at 37 °C for 1 h. After washing three times, 50 μL of Substrate A and 50 μL of Substrate B were added to each well, followed by incubation in the dark at 37 °C for 10 min; then, 50 μL of stop solution was added. Finally, absorbance was measured at 450 nm within 15 min using a Biotek (Biotek, Winooski, VT, USA) reader, and the Glu content in the serum was calculated based on a standard curve.

### 2.8. Analysis of PKC Activity and Signaling Molecules

The activity of protein kinase C (PKC) and the concentrations of phospholipase C (PLC), diacylglycerol (DAG), inositol trisphosphate (IP3), and calcium ions (Ca^2+^) were measured using ELISA kits. These kits included the Shellfish PKC ELISA Kit (Mlbio, Shanghai, China), Shellfish PLC ELISA Kit (Mlbio, Shanghai, China), Shellfish DAG/DG ELISA Kit (Mlbio, Shanghai, China), and the IP3 ELISA Kit (Elabscience, Wuhan, China). The procedures were followed as per the manufacturer’s instructions. The kits use a double-antibody sandwich ELISA method with a TMB substrate for color development. TMB is converted to a blue color under peroxidase catalysis, and the color finally turns yellow under acidic conditions. The intensity of the color is positively correlated with enzyme activity or concentration in the sample.

Calcium ions were detected by complexing with o-cresolphthalein under alkaline conditions to form a purple complex. The calcium ion content was quantified by measuring the absorbance at 575 nm using a spectrophotometer. The Calcium Content Colorimetric Assay Kit (Beyotime Biotechnology, Shanghai, China) was used for calcium ion detection, and the procedure was followed according to the kit instructions. Absorbance values were read at 575 nm, and calcium ion content was calculated.

### 2.9. Statistical Analysis

All data are presented as mean ± standard deviation (SD), and statistical analysis was performed using OriginPro 8.0 software. Inter-group differences were analyzed using one-way ANOVA, followed by Tukey’s post hoc test. Differences were considered statistically significant when *p* < 0.05. Groups with different letters (a, b, c, d) indicated statistically significant differences, while groups sharing the same letter were not significantly different.

## 3. Results

### 3.1. Response of CgmGluR5 to V. splendidus and Glu Treatment

**Figure 1:**
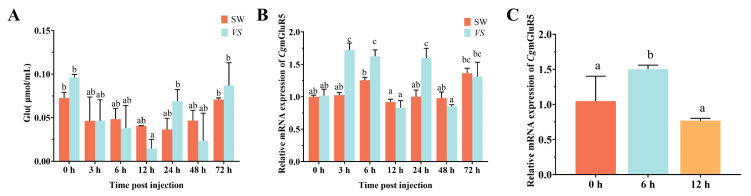
*CgmGluR5* is responsive to treatment with *V. splendidus* and Glu. (**A**) Changes in Glu concentration in the serum at different time points (0, 3, 6, 12, 24, 48, and 72 h) following *V. splendidus* (*VS*) treatment and sterile seawater (SW) treatment. (**B**) Expression levels of *CgmGluR5* in hemocytes at different time points (0, 3, 6, 12, 24, 48, and 72 h) following *V. splendidus* (*VS*) treatment and sterile seawater (SW) treatment. (**C**) Expression levels of *CgmGluR5* in hemocytes at different time points (0, 6, and 12 h) after Glu incubation. Error bars indicate mean ± standard deviation (n = 3). Significant differences between groups are indicated by different letters (a, b, c), with *p* < 0.05.

To investigate the regulatory role of *CgmGluR5* in immune responses, its expression was analyzed following treatment with *V. splendidus* (*VS*) and glutamate (Glu). Glu content decreased upon *V. splendidus* injection, reaching a minimum of 0.0146 μmol/L at 12 h, representing 0.36 times the level observed in the control (sterile seawater group), and subsequently returned to baseline (Figure 1A). *CgmGluR5* mRNA levels in hemocytes peaked at 3 h post-treatment, showing a 1.68-fold increase compared to the control (3 h SW vs. 3 h *VS*: *p* < 0.0001), with additional significant elevations at 6 h (1.29-fold) and 24 h (1.59-fold) (6 h SW vs. 6 h *VS*: *p* = 0.0085; 24 h SW vs. 24 h *VS*: *p* < 0.0001) (Figure 1B). Following Glu incubation, *CgmGluR5* mRNA levels reached a peak at 6 h, corresponding to a 1.43-fold increase relative to the control (0 h vs. 6 h: *p* = 0.0311), before returning to baseline at 12 h (Figure 1C). These temporal patterns of *CgmGluR5* expression in response to *V. splendidus* and Glu suggest its involvement in modulating immune responses in *C. gigas* hemocytes.

### 3.2. Response of CgmGluR5 to Glu Treatment

**Figure 2:**
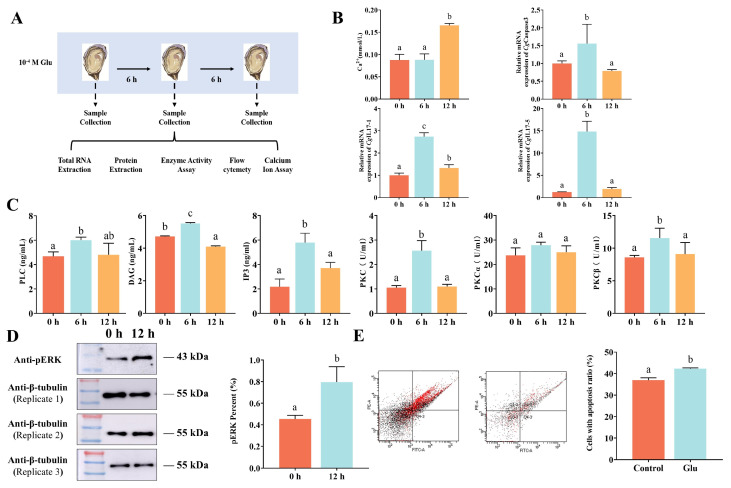
Analysis of key downstream molecule expression levels of *CgmGluR5* in hemocytes following Glu treatment. (**A**) Schematic diagram of the Glu treatment experiment. (**B**) Changes in the levels or expression of Ca^2+^, *CgCaspase3*, *CgIL17-1*, and *CgIL17-5* in hemocytes at different time points (0, 6, and 12 h) after Glu treatment. (**C**) Changes in the levels or activity of PLC, DAG, IP3, PKC, PKCα, and PKCβ in hemocytes at different time points (0, 6, and 12 h) after Glu treatment. (**D**) Changes in the expression level of pERK protein in hemocytes after Glu treatment, with β-Tubulin as the internal control; panels represent three independent β-Tubulin Western blots (Replicate 1, Replicate 2, Replicate 3); ImageJ (version 1.53t) was used for statistical analysis of pERK protein expression levels. (**E**) Percentage of apoptotic-positive hemocytes in the total hemocyte population after Glu treatment. Error bars represent the mean ± standard deviation (n = 3). Significant differences between groups are indicated by different letters (a, b, c), *p* < 0.05.

The influence of *CgmGluR5* on immune molecule expression was examined by analyzing its regulation of *CgIL17-1* and *CgIL17-5*. Six hours after glutamate (Glu) incubation, levels and activities of PLC, DAG, IP3, and PKC significantly increased by 1.29-, 1.29-, 2.65-, and 2.42-fold relative to the control group (0 h vs. 6 h: *p* = 0.0065, *p* < 0.0001; *p* = 0.0011; *p* = 0.0084), returning to baseline by 12 h (Figure 2C). This trend indicates that Glu activates the phosphoinositide pathway through *CgmGluR5*. Ca^2+^ levels rose to 0.17 mmol/L, a 1.89-fold increase compared to controls at 12 h. At 6 h, the mRNA expression of *CgIL17-1* was significantly upregulated (2.73-fold, 0 h vs. 6 h: *p* < 0.0001), while *CgIL17-5* and *CgCaspase3* mRNA levels peaked at 14.82- and 1.56-fold in control levels, respectively (Figure 2B). Western blotting confirmed a 1.76-fold increase in pERK protein levels at 12 h (Figure 2D). Flow cytometry showed that hemocyte apoptosis in the Glu-treated group rose to 42.3%, 1.14-fold higher than in the control (Figure 2E). These results demonstrate that *CgmGluR5*, through activation of the phosphoinositide pathway, modulates the expression of *CgIL17-1*, *CgIL17-5*, and *CgCaspase3,* elevates pERK levels, and promotes hemocyte apoptosis, thereby contributing to immune regulation.

**Figure 3:**
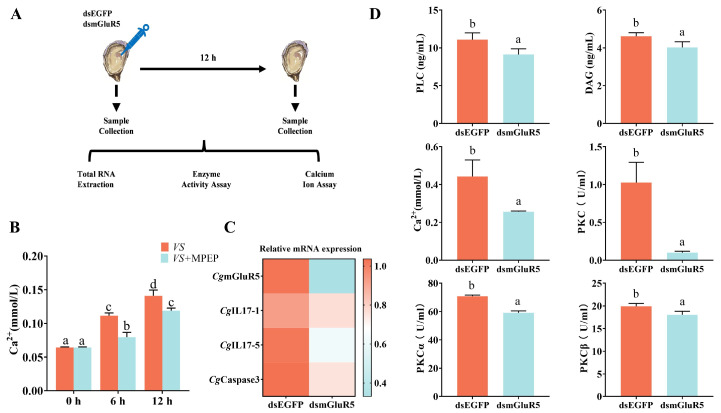
Expression levels of key downstream molecules of *CgmGluR5* in hemocytes after dsRNA injection. (**A**) Schematic of the interference experiment. (**B**) The change in Ca^2+^ levels after injection of the mGluR5-specific inhibitor MPEP. (**C**) Changes in the expression of *CgmGluR5*, *CgIL17-1*, *CgIL17-5*, and *CgCaspase3* in hemocytes 12 h after *CgmGluR5* interference. (**D**) Levels or activity changes of PLC, DAG, Ca^2+^, PKC, PKCα, and PKCβ in hemocytes 12 h after *CgmGluR5* interference. Significant differences between groups are indicated by different letters (a, b, c, d), *p* < 0.05.

The regulatory role of *CgmGluR5* in modulating *IL-17* expression through the phosphoinositide pathway was examined using dsRNA-mediated interference. Twelve hours post-injection, the activity levels of PLC, DAG, Ca²⁺, and PKC significantly decreased to 0.82-, 0.87-, 0.58-, and 0.10-fold of the dsEGFP control group, respectively (dsEGFP vs. dsmGluR5: *p* = 0.0065, *p =* 0.0466, *p* = 0.0209, *p* = 0.0005) (Figure 3D). The activities of PKCα and PKCβ also reduced to 0.83- and 0.91-fold of the control group (dsEGFP vs. dsmGluR5: *p* = 0.0002, *p* = 0.0304) (Figure 3D). Quantitative real-time PCR revealed that *CgmGluR5* mRNA levels decreased to 0.32-fold, with concurrent reductions in *CgCaspase3*, *CgIL17-1*, and *CgIL17-5* expressions to 0.72-, 0.82-, and 0.60-fold of control levels (dsEGFP vs. dsmGluR5: *p* < 0.0001, *p =* 0.2177, *p* = 0.0009) (Figure 3C). After the injection of MPEP, Ca²⁺ levels decreased at both 6 h and 12 h to 0.71-fold and 0.84-fold that of the control group, respectively (6 h *VS* vs. 6 h *VS* + MPEP: *p* < 0.0001; 12 h *VS* vs. 12 h *VS* + MPEP: *p* = 0.0027) (Figure 3B). These findings indicate that *CgmGluR5* may regulate immune factor expression, including *CgIL17-1* and *CgIL17-5*, through the modulation of PLC, DAG, Ca²⁺, and classical PKC activity.

### 3.3. CgmGluR5 Regulation of IL-17 Through PLC and PKC

**Figure 4:**
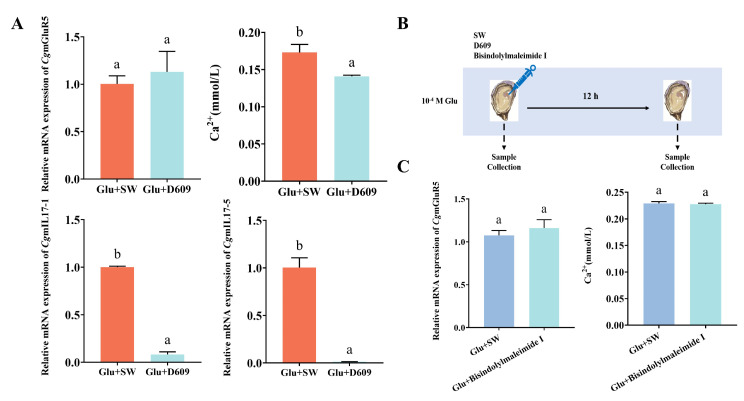
Expression levels of key downstream molecules of *CgmGluR5* in hemocytes after injection of D609 and Bisindolylmaleimide I. (**A**) Changes in expression or levels of *CgmGluR5*, Ca^2+^, *CgIL17-1*, and *CgIL17-5* in hemocytes 12 h after PLC inhibition. (**B**) Schematic of the inhibition experiment. (**C**) Changes in expression or levels of *CgmGluR5* and Ca^2+^ in hemocytes 12 h after PKC inhibition. Significant differences between groups are indicated by different letters (a, b), with *p* < 0.05.

The role of *CgmGluR5* in immune modulation was examined by measuring *CgmGluR5*, *CgIL17-1*, and *CgIL17-5* expression in hemocytes following PLC inhibitor D609 injection. Results showed that the relative expression of *CgmGluR5* mRNA slightly increased 12 h after injection but without a significant change. By contrast, the relative mRNA expression levels of *CgIL17-1* and *CgIL17-5* significantly decreased (Glu + SW vs. Glu + D609: *p* < 0.0001, *p* < 0.0001) to 0.08-fold and 0.01-fold of the control group, respectively (Figure 4A). Using a Ca²⁺ assay kit, we measured changes in Ca^2+^ content in hemocytes of *C. gigas* after injection of the PLC inhibitor D609 and PKC inhibitor Bisindolylmaleimide I. Our R = results indicated that Ca^2+^ levels significantly decreased to 0.14 mmol/L 12 h after D609 injection (Glu + SW vs. Glu + D609: *p* = 0.0070), reaching 0.81-fold of the Glu + SW group (Figure 4A). By contrast, there was no significant change in Ca^2+^ levels 12 h after Bisindolylmaleimide I injection (Figure 4C). *CgmGluR5* expression levels showed no significant change 12 h after injection of either D609 or Bisindolylmaleimide I (Figure 4A,C). These findings suggest that *CgmGluR5* may regulate Ca^2+^ signaling by affecting PLC, which subsequently influences classical PKC and indirectly modulates the expression of immune factors such as *CgIL17-1* and *CgIL17-5*.

### 3.4. Modulation of Hemocyte Subpopulation Proportions by CgmGluR5

**Figure 5:**
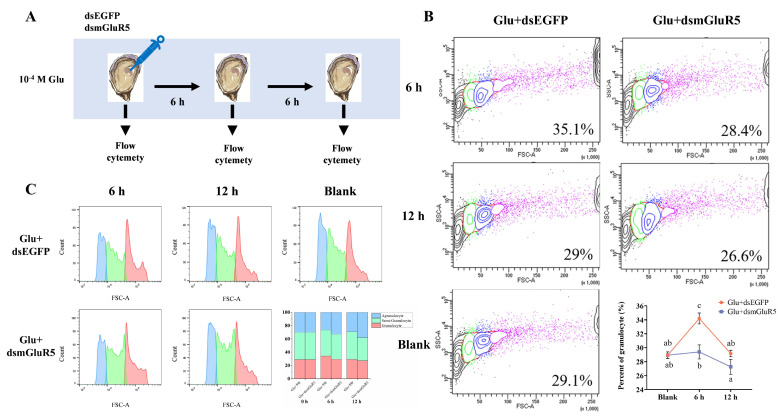
Proportions of the three hemocyte subpopulations in total hemocytes after dsRNA injection. (**A**) Diagram of the cell typing experiment. (**B**) Proportional statistics of granulocytes among total hemocytes at different time points (0, 6, and 12 h) after Glu + dsRNA combined treatment and following Glu treatment after interfering with *CgmGluR5* expression. (**C**) Proportional statistics of the three hemocyte types at different time points (0, 6, and 12 h) after Glu + dsRNA combined treatment and following Glu treatment after interfering with *CgmGluR5* expression. Significant differences between groups are indicated by different letters (a, b, c), *p* < 0.05.

To investigate the effect of *CgmGluR5* on hemocyte processes, the proportion of three hemocyte subpopulations was determined by flow cytometry after silencing *CgmGluR5* with its double-stranded RNA (dsRNA). Six hours post-injection, compared to the Glu + dsEGFP group, the proportion of granulocytes in total hemocytes in the Glu + dsmGluR5 group decreased significantly from 34.2% to 29.4% (6 h Glu + dsEGFP vs. 6 h Glu + dsmGluR5: *p* < 0.0001), which was 0.86 times that of the Glu + dsEGFP group (Figure 5B). After 12 h of injection, the proportion of granulocytes in total hemocytes decreased from 29.2% to 27.3%, which was 0.93 times that of the Glu + dsEGFP group (Figure 5B). These results show that inhibiting *CgmGluR5* expression leads to a reduction in the proportion of granulocytes, indicating that *CgmGluR5* acts as a regulatory factor in hemocyte proliferation and differentiation during immune responses.

## 4. Discussion

This study provides novel evidence that metabotropic glutamate receptor 5 (*mGluR5*) in the Pacific oyster functions as a critical regulator within the glutamate-mediated immune system. Previous studies have demonstrated that Pacific oysters adjust glutamate levels in response to immune challenges, particularly during the early phases of immune activation, by promoting glutamate secretion to facilitate pathogen clearance [23]. Despite these findings, the activation mechanisms, associated signaling pathways, and functional roles of the glutamatergic system in invertebrate immunity remain inadequately characterized. The present study reveals that *CgmGluR5* mediates immune regulation in *C. gigas* by responding to bacterial infection and glutamate treatment, thereby activating downstream signaling cascades and modulating the expression of inflammatory mediators. It also influences apoptotic pathways, ultimately contributing to immune defense processes.

Glutamate has been reported to regulate cytokine secretion and modulate immune responses in vertebrates [24]. For instance, glutamate released by dendritic cells inhibits *IL-6* production through *mGluR5* receptors expressed on resting human T cells [25,26]. In the present study, we observed a decrease in serum glutamate levels in *C. gigas* following *V. splendidus* treatment, accompanied by upregulation of *CgmGluR5* expression. This initial decrease in glutamate could reflect its consumption or redistribution during early immune response, potentially due to increased uptake by immune cells or its use in metabolic processes. Interestingly, serum glutamate levels subsequently increased, possibly indicate a compensatory mechanism or the release of glutamate from cells as part of immune activation. In addition, a similar trend was observed in the sterile water control group. This observation could be due to non-specific factors such as the stress response caused by the experimental procedure itself or fluctuations in cellular activity during the treatment period, which could lead to changes in glutamate levels even in the absence of pathogen-specific stimulation. Additionally, *CgmGluR5* expression was significantly elevated upon glutamate treatment. In vertebrate immune cells, *mGluR5* mediates various immune functions, including inhibition of T cell proliferation and modulation of cytokine secretion [27]. In invertebrates, *mGluR5* has been identified in the Pacific oyster, where it influences calcium homeostasis and contributes to immune responses by regulating *CALM1* [2]. These findings indicate that glutamate consumption increases during immune activation and may contribute to immune defense mechanisms through *CgmGluR5*. It also suggests that glutamate, functioning as a signaling molecule, could be released upon pathogen invasion, activating *CgmGluR5* and initiating downstream signaling pathways to trigger immune responses.

Metabotropic glutamate receptor 5 has been shown to participate in immune responses by activating the Akt/PI3K signaling pathway, which regulates *NF-κB* and subsequently upregulates pro-inflammatory cytokines such as *IL-6* and *IL-8* [28]. However, the current study did not explore this pathway; rather, our focus was on the role of *CgmGluR5* in regulating immune cell activity and cytokine expression. In this study, Glu treatment led to a significant increase in the mRNA expressions of *CgIL17-1* and *CgIL17-5* in *C. gigas* hemocytes, an effect that was abolished following *CgmGluR5* interference. This finding indicates that *CgmGluR5* is critical for the regulation of *CgIL17-1* and *CgIL17-5*. Additionally, *IL17-1* and *IL17-5* are known to play pivotal roles in inflammatory responses, apoptosis, and immune cell interactions [29,30]. Therefore, *CgmGluR5*-mediated upregulation of these cytokines likely facilitates immune cell activation and enhances immune defense capabilities in oysters. Furthermore, Glu treatment significantly increased the levels of PLC, PKC, DAG, Ca^2+^, and IP3 in hemocytes and activated pERK protein. This result suggests that *CgmGluR5* activates the phosphoinositide pathway, thereby modulating intracellular Ca²⁺ signaling. The inhibition of *CgmGluR5* expression or blockade of PLC and PKC pathways led to a marked decrease in inflammatory cytokine expression, further confirming the regulatory role of *CgmGluR5* in inflammatory cytokine expression through the phosphoinositide pathway.

Although glutamate can be toxic at high concentrations, it still induces an immune response, suggesting it plays a role in regulating immune cell activity, possibly through mechanisms like apoptosis. Apoptosis is a form of programmed cell death that serves a fundamental role in immune defense mechanisms [31,32]. During an immune response, apoptosis helps eliminate infected or damaged cells, maintaining tissue homeostasis and preventing chronic inflammation. This study demonstrated that *CgmGluR5* may influence hemocyte apoptosis through the regulation of *CgIL17-1* and *CgIL17-5* expression. Following Glu treatment, significant upregulation of *CgmGluR5*, *CgIL17-1*, and *CgIL17-5* was observed in hemocytes, accompanied by increased mRNA levels of the apoptosis-related gene *CgCaspase3* and a heightened hemocyte apoptosis rate. *IL17-1* and *IL17-5* are recognized for their pro-apoptotic effects in immune cells, particularly under inflammatory conditions induced by infections, where they promote cell death by activating apoptosis-related signaling pathways [14,33]. These findings suggest that *CgmGluR5* may modulate hemocyte apoptosis by upregulating *CgIL17-1* and *CgIL17-5*, thereby activating genes such as *CgCaspase3*. Previous studies have also shown that glutamate activates calcium channels through NMDA receptors, leading to increased ROS production and *caspase-3*-dependent apoptosis [34]. This finding implies that *CgmGluR5* participates in apoptosis regulation through *CgIL17-1* and *CgIL17-5*, although further research is needed to clarify the precise mechanisms involved.

Granulocytes serve as primary effector cells in molluscan immune responses, primarily protecting against pathogens through phagocytosis and bactericidal activity [21,35]. Additionally, they enhance immune responses by secreting cytokines and reactive oxygen species, contributing significantly to pathogen clearance [35,36]. In this study, hemocytes treated with Glu exhibited significant upregulation of *CgmGluR5*, *CgIL17-1*, and *CgIL17-5* (*p* < 0.05). Inhibition of *CgmGluR5* expression followed by Glu treatment led to marked downregulation of *CgIL17-1* and *CgIL17-5* (*p* < 0.05) and a notable decrease in the proportion of granulocytes in the hemolymph (*p* < 0.05). Previous studies have demonstrated that *CgIL17-1* plays a role in inflammatory regulation and hemocyte proliferation within the innate immune response of Pacific oysters [37,38]. In vertebrates, *mGluR5* activation stimulates the ERK and Akt signaling pathways, thereby promoting cell proliferation and differentiation, including roles in neurogenesis and cellular maturation [3,39,40]. These observations suggest that Glu, acting through *CgmGluR5*, upregulates *CgIL17-1* and *CgIL17-5* expression, thereby influencing hemocyte differentiation and enhancing granulocyte production. Given the critical function of granulocytes in molluscan immunity, activation of *CgmGluR5* likely strengthens the immune defense mechanisms of *C. gigas*.

## 5. Conclusions

Neurotransmitter systems play a critical role in the immune regulation of marine invertebrates, drawing increasing attention for their importance in host defense. This study reveals that *CgmGluR5* mediates sustained regulation of the immune responses of the Pacific oyster through modulation of the phosphoinositide pathway. The findings indicate that *CgmGluR5* responds to bacterial stimulation and glutamate treatment, orchestrating long-term immune responses by modulating the expression of critical immune factors, including *CgIL17-1* and *CgIL17-5*, as well as regulating hemocyte differentiation, apoptosis, and granulocyte production. This regulatory mechanism underscores the importance of the glutamate system in molluscan innate immunity. By elucidating the interactions of *CgmGluR5* with immune pathways, this research deepens our understanding of neuroimmune regulation in invertebrates in sustained immune regulation. It also provides a theoretical basis for improving disease management strategies in aquaculture.

## Data Availability

All study data are included in the article. For seeking other information and materials that are related to this project, please contact the corresponding author.

## Supplementary Materials

The following supporting information can be downloaded at: https://www.mdpi.com/article/10.3390/cells14060438/s1, Table S1. Primer sequences used in this study.

## Abbreviations

SW: seawater; *VS*: *Vibrio splendidus*.
