# Supplementary material for: Long-Term Regulation of IL-17 Expression in Pacific Oyster Hemocytes by mGluR5 Through the Phosphoinositide Pathway
Source: Cells. 2025 Mar 14;14(6):438. doi: 10.3390/cells14060438 (PMC11941601; doi:10.3390/cells14060438)
Supplement: Supplementary file 1 [file cells-14-00438-s001.zip › cells-3362092-supplementary.pdf]

Supplementary Table S1. Primer sequences used in this study.

**Primers**	**Sequence (5′-3′)**
**RNAi Primers**	
ds*CgmGluR5*-F	CCCAAGCTTCGAGGAAAACGTAGCTG-GAG
ds*CgmGluR5*-R	CTAGCTAGCAGAGCTGACCAGTCTCCCAA
dsEGFP-F	CCCAAGCTTACGTAAACGGCCACAAGTTC
dsEGFP-R	CTAGCTAGCTGTTCTGCTGGTAGTGGTCG
**qRT-PCR Primers**	
CgmGluR5-RT-F	CTTTGCCCTGAGATGCTTCC
CgmGluR5-RT-R	TATCATACCTTGCTGGCGGA
CgCaspase3-RT-F	CGGGAAATTACGGGGAGTTG
CgCaspase3-RT-R	TCTTCGGAGGATACAGAGGG
CgIL17-1-RT-F	ACTGAGGCTCGATGCAAGTG
CgIL17-1-RT-R	AGCCTTCTTGCTTCATGTGG
CgIL17-5-RT-F	CGTCCTTGCCTTACTGACTAGA
CgIL17-5-RT-R	TGTCGTTGTCCTCTACCATGAT
CgEF-1-RT-F	AGTCACCAAGGCTGCACAGAAAG
CgEF-1-RT-R	TCCGACGTATTTCTTTGCGATGT
